# ADC-based radiomics for risk stratification in prostate cancer: a clinical decision support study

**DOI:** 10.3389/fonc.2026.1885686

**Published:** 2026-08-31

**Authors:** Kun Zhang, Jiajun Zhang, Yangguang Yuan, Zhijing Chen, Yifeng Du, Hanlin Liu

**Affiliations:** 1 Department of Radiology, The Third Affiliated Hospital (The Affiliated Luohu Hospital) of Shenzhen University, Shenzhen, Guangdong, China; 2 Department of Radiology, National Cancer Center/National Clinical Research Center for Cancer/Cancer Hospital & Shenzhen Hospital, Chinese Academy of Medical Sciences and Peking Union Medical College, Shenzhen, Guangdong, China

**Keywords:** apparent diffusion coefficient, diagnostic support, machine learning, magnetic resonance imaging, prostate cancer, radiomics

## Abstract

**Introduction:**

Reliable differentiation between malignant and benign prostate lesions remains a critical challenge in clinical practice, particularly in settings with limited access to expert interpretation of multiparametric MRI (mpMRI). We investigated whether a streamlined radiomics framework based solely on apparent diffusion coefficient (ADC) imaging could provide accurate, reproducible, and interpretable diagnostic support for prostate cancer detection.

**Methods:**

We retrospectively analyzed ADC images from 198 men, including 121 patients with histologically confirmed prostate cancer and 77 patients without malignancy, acquired between 2020 and 2025. Following image resampling and standardization, 107 radiomic features were extracted and subjected to hierarchical feature selection using recursive feature elimination followed by LASSO regression. Four discriminative features were retained to train support vector machine, random forest, logistic regression, and gradient boosting decision tree (GBDT) classifiers. Model performance was evaluated in independent training and testing cohorts using accuracy, AUC, precision, recall, F1 score, and specificity.

**Results:**

The GBDT model demonstrated the most robust performance, achieving AUC of 0.81 (95% CI: 0.75–0.87) in the training cohort and 0.78 (95% CI: 0.72–0.85) in the testing cohort. In the testing cohort, the model achieved an accuracy of 0.76 (95% CI: 0.69–0.84), a recall of 1.00 (95% CI: 1.00–1.00), an F1 score of 0.86 (95% CI: 0.80–0.92), and a specificity of 0.73 (95% CI: 0.62–0.88). The selected radiomic features reflected image energy, textural irregularity, and intralesional heterogeneity and were consistently associated with malignant pathology.

**Discussion:**

A minimal-feature radiomics model derived exclusively from ADC images showed promising diagnostic performance for differentiating malignant from benign prostate lesions. By avoiding reliance on multiparametric imaging, this streamlined approach may provide a practical, reproducible, and resource-efficient strategy for supporting prostate cancer diagnosis, particularly in settings where expert mpMRI interpretation is limited. Further multicenter validation, integration of clinical variables, and incorporation of explainable artificial intelligence approaches are warranted to establish its robustness and enhance its translational potential.

## Introduction

Prostate cancer (PCa) is the second most commonly diagnosed cancer in men and represents a major threat to male health worldwide (1, 2). It is the fifth leading cause of cancer-related mortality among men globally (3). Early detection of PCa is critical for improving patient outcomes, while accurate subtype classification and staging are essential for guiding treatment decisions (4, 5). Therefore, reliable diagnosis of PCa has substantial clinical value. Since 2020, the European Association of Urology has recommended prostate biopsy as a standard approach to obtain more accurate information on tumor aggressiveness (6). Although biopsy provides valuable pathological confirmation, it is invasive and may adversely affect treatment options and patient prognosis (7). Moreover, biopsy samples are limited to specific sampling sites and cannot fully capture the spatial heterogeneity of the tumor, which may lead to incomplete assessment of disease characteristics (8, 9).

Within multiparametric MRI (mpMRI), diffusion-weighted imaging and its quantitative derivative, the apparent diffusion coefficient (ADC), capture restricted water diffusion associated with increased cellularity and stromal alterations (10). These features have made ADC a valuable tool for PCa screening and treatment planning (4). In line with this, the National Comprehensive Cancer Network (NCCN) strongly recommends mpMRI as an intermediate step between elevated prostate-specific antigen levels and prostate biopsy (11, 12). Despite these advantages, several challenges remain. First, PCa assessment based on ADC relies heavily on radiologist expertise, limiting its reliability in settings with constrained medical resources and leading to substantial inter- and intra-observer variability (13). Second, benign and malignant lesions often exhibit highly similar imaging appearances, making accurate differentiation difficult even for experienced clinicians.

In the era of rapid advances in computer-aided diagnosis, radiomics has emerged as a powerful approach by extracting high-dimensional features across multiple imaging sequences, including handcrafted and deep features (14–17). These features are combined with conventional statistical analyses and relatively simple machine learning models to construct diagnostic frameworks (18). Radiomics-based methods have demonstrated strong performance across a range of clinical tasks, including adjuvant therapy decision (19), histological classification (20), and prognosis prediction (21), in some cases outperforming expert radiologist assessment (21). Radiomics also offers high reproducibility and requires substantially fewer computational resources than end-to-end deep learning approaches, which enhances its practicality and generalizability (18). Moreover, carefully designed features—such as gray-level co-occurrence matrices and entropy-based measures—can capture subtle imaging patterns that are not discernible to the human eye (22). This capability provides a promising means to distinguish benign from malignant lesions that exhibit highly similar morphological characteristics.

The aim of this study was to develop a machine learning model based on ADC images for distinguishing benign from malignant prostate lesions. ADC was selected due to its established biological correlation with tumor cellularity and its robustness across clinical scanners. We constructed a diagnostic pipeline by extracting interpretable handcrafted features from ADC images and integrating them with several widely used machine learning algorithms. Through feature reduction and model optimization, the proposed approach achieved effective PCa detection, with an AUC of 0.78. In addition, model interpretability analysis identified multiple features strongly associated with malignancy, providing imaging-based insights that may support further investigation of PCa characteristics.

## Materials and methods

### Study design and oversight

This single-center, retrospective diagnostic-modeling study used de-identified ADC images from men referred for prostate MRI between January 2020 and March 2025. All procedures adhered to the Declaration of Helsinki and were approved by the institutional review board of The Third Affiliated Hospital (The Affiliated Luohu Hospital) of Shenzhen University under protocol 2025-LHQRMYY-KYLL-025, with a waiver of informed consent granted for secondary analysis of anonymized data.

### Participants

**Figure 1:**
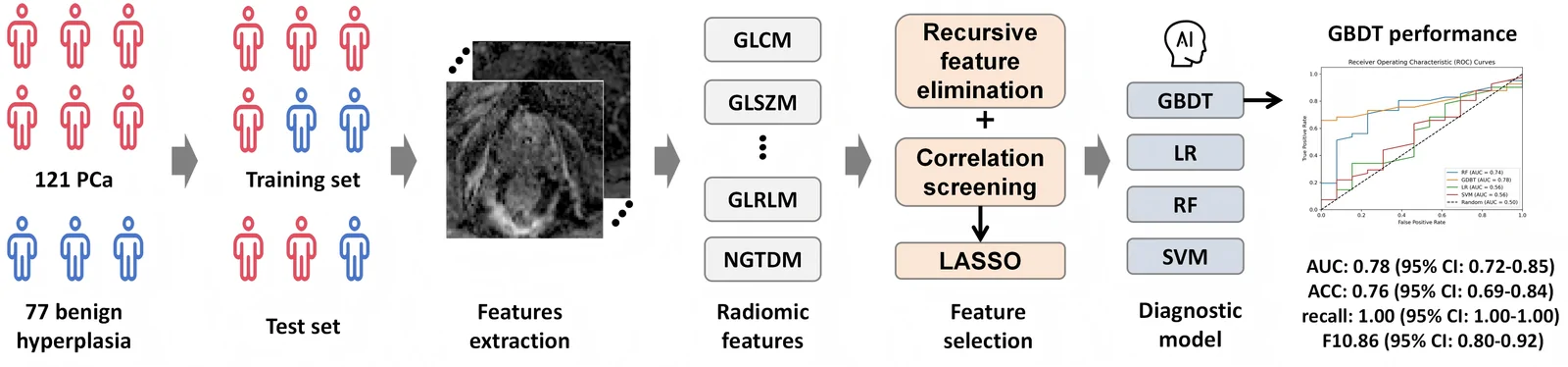
Schematic of the radiomics workflow. A total of 198 individuals (121 prostate cancer patients and 77 volunteers with benign prostatic hyperplasia) were randomly divided into a training set and a test set in an 80:20 ratio. Next, feature extraction and feature selection were performed on the ADC images, and finally, diagnostic models were constructed using various machine learning models.

Included cases were men aged 18 to 95 who underwent a complete prostate MRI within four weeks prior to biopsy and were eligible for prospective PI-RADS assessment; who had complete PSA-related laboratory test data obtained prior to biopsy and before any treatment; and who received a histopathological diagnosis through systematic biopsy (including targeted sampling). The non-PCa cohort included men without urinary tract malignancies who underwent the same MRI scanning protocol; benign prostatic hyperplasia was eligible for inclusion. Exclusion criteria include: a history of prostate cancer treatment, including radiation therapy, androgen deprivation therapy, prostate surgery, or systemic anticancer therapy; acute prostatitis or urinary tract infection at the time of PSA measurement; incomplete clinical, laboratory, or imaging data; MRI image quality insufficient for PI-RADS interpretation; and a known history of prostate cancer. The final analytic set comprised 198 individuals, including 121 with PCa and 77 without malignancy, randomly partitioned at the patient level into an 80:20 training–testing split to preserve an untouched hold-out set for validation and to avoid information leakage from model selection decisions. **Figure 1** shows schematic of the radiomics workflow.

### MRI acquisition

**Figure 2:**
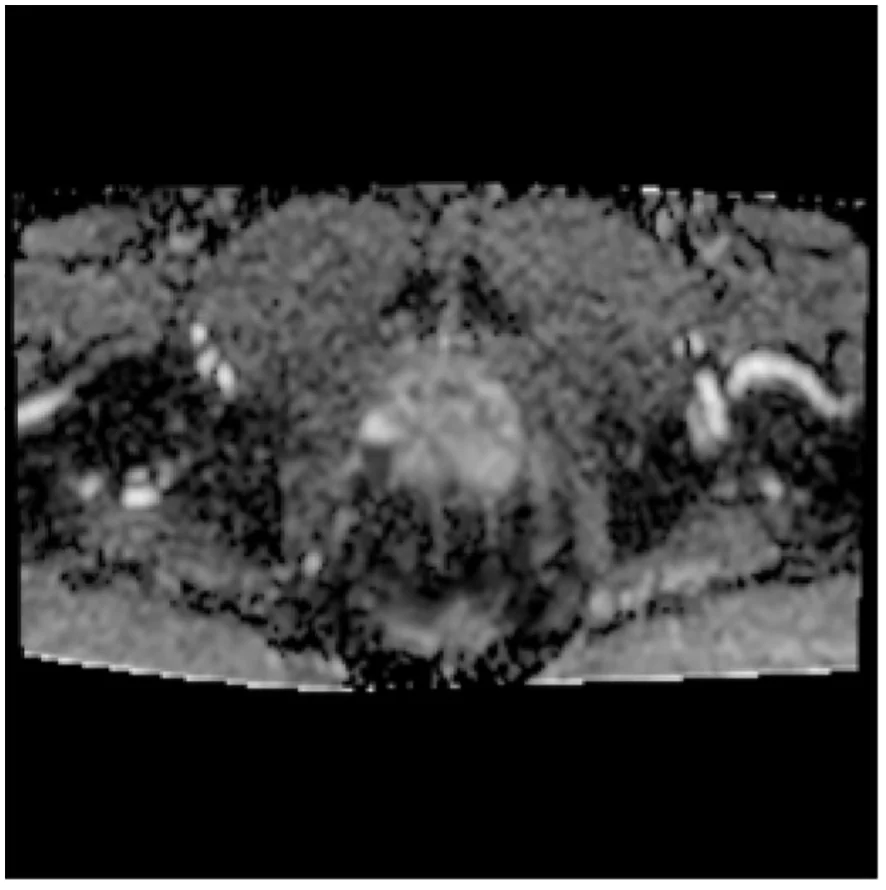
Show MRI image of a patient on ADC. Image preprocessing and analysis masks.

Imaging was performed on two clinical scanners: a 1.5-T Siemens MAGNETOM Aera and a 3.0-T GE SIGNA Architect. Representative diffusion parameters for Siemens were repetition time 5900 ms, echo time 72 ms, slice thickness 3.0 mm with 0.6-mm interslice gap, a 128×128 matrix, and a 22×22 cm field of view. For GE, repetition time was 5440 ms, echo time 86.9 ms, slice thickness 3.0 mm with a 1-mm gap, a 140×70 matrix, and a 26×15.6 cm field of view. ADC maps were reconstructed from multiple b-values of 50, 800, 1400, and 2000 s/mm². Acquisition protocols were harmonized to the extent possible across platforms, and images failing prespecified quality criteria were removed prior to analysis (**Figure 2**). ComBat Harmonization and N4 Bias Correction are used to calibrate the scanner field bias and model-specific variations, respectively.

To mitigate inter-scanner heterogeneity in voxel geometry, all ADC volumes were resampled to isotropic 1×1×1 mm spacing using linear interpolation. Feature values were standardized by z-scoring within the training data, and the resulting scaling parameters were applied to the test set to prevent leakage. A standardized prostate-region analysis mask was used to support an annotation-light workflow, thereby avoiding manual lesion tracing in the primary analysis while maintaining reproducibility of the feature extraction process.

### Radiomic feature extraction

The proposed method does not require PCa segmentation of MRI images; instead, it performs feature extraction on the complete ADC images. Radiomics features were extracted using PyRadiomics (version v3.1.0) implemented with SimpleITK (version v2.4.0). The original CT images and corresponding segmentation masks were used as inputs. No additional intensity normalization or image resampling was applied before feature extraction. Radiomics features were calculated using the default PyRadiomics parameter settings (binWidth: 25), including the default gray-level discretization strategy and default feature extraction configuration. The extracted features included first-order statistics, shape features, and texture features derived from gray-level co-occurrence matrix (GLCM), gray-level run length matrix (GLRLM), gray-level size zone matrix (GLSZM), gray-level dependence matrix (GLDM), and neighboring gray-tone difference matrix (NGTDM) (23–26). Prior stability checks indicated that LHL-band (Low-High-Low frequency band) wavelet variables were less robust to minor perturbations and contributed little discriminative signal, and they were therefore excluded *a priori* to enhance downstream stability and parsimony. In total, 107 features were retained for subsequent selection procedures.

### Feature selection

Dimensionality reduction proceeded in two stages conducted strictly within the training cohort. An initial redundancy pruning combined recursive feature elimination with correlation screening to reduce multicollinearity while preserving complementary signal. A sparsity-inducing step based on least absolute shrinkage and selection operator (LASSO) regression then identified a compact signature that balanced bias and variance under 10-fold cross-validated tuning of the regularization parameter. The final signature contained four non-redundant predictors, including first-order Energy, GLCM Cluster Prominence, GLDM Dependence Non-Uniformity, and GLRLM Run-Length Non-Uniformity, that capture voxel-intensity magnitude, higher-order textural asymmetry, and heterogeneity in local dependence and run-length patterns (27).

### Model development

Four conventional classifiers were trained on the selected features using scikit-learn pipelines that encapsulated preprocessing, selection, and learning to prevent inadvertent data leakage. Logistic regression (LR) (28) employed the liblinear solver; the support-vector machine (SVM) (29)used a radial-basis kernel with hyperparameters tuned within a constrained grid in the training set; and the ensemble models named random forest (RF) (30) and gradient boosting decision trees (GBDT) (31) used 50 estimators each based on empirical calibration in the training partition. All modeling choices, including hyperparameters, were finalized without reference to the test data.

### Performance evaluation

Discrimination was summarized by the area under the receiver-operating-characteristic (ROC) curve on the independent test set. Accuracy, precision, recall, and F1 score provided complementary threshold-dependent summaries at the default operating point, while ROC analysis offered a threshold-free view of model behavior. The primary analytic focus was whether a minimal ADC-only signature coupled with GBDT could sustain high sensitivity without sacrificing overall accuracy when compared with LR, RF, and SVM baselines.

### Reproducibility, bias safeguards, and ethics

All selection and tuning were confined to the training cohort, and the hold-out test set was reserved for a single final evaluation. The fixed resampling and scaling procedures were applied identically across cohorts, and the compact four-feature signature was chosen to reduce variance and improve replicability in modest sample sizes. Data were de-identified prior to analysis, with access restricted to study personnel under institutional governance; ethical approvals and waivers are detailed above.

## Results

### Baseline characteristics

**Table 1** summarizes the demographic and clinical characteristics of the entire study cohort. Among the 198 enrolled patients, 77 were classified as negative (median age: 65.71 years; median PSA: 6.66 ng/mL; median fPSA: 1.44 ng/mL; median fPSA/PSA ratio: 0.19), while 121 were classified as positive (median age: 70.24 years; median PSA: 16.52 ng/mL; median fPSA: 1.97 ng/mL; median fPSA/PSA ratio: 0.13). Statistically significant differences were observed between the two groups across all evaluated variables (P < 0.05).

**Table 1:** Characteristics of the participants at baseline.

Characteristics	Benign	Malignant	*t/Z*	*P*
Patients in total	77	121		
Age, Mean ± SD (years)	65.74 ± 8.81	70.24 ± 8.13	3.881	<0.001
PSA (ng/mL)	6.66(4.89, 13.62)	16.52(8.33, 37.76)	-5.614	<0.001
fPSA (Q1,Q3) (ng/mL)	1.44(0.91, 2.59)	1.97(1.11, 4.70)	-2.341	0.019
fPSA/PSA	0.19(0.15, 0.24)	0.13(0.09, 0.17)	-5.354	<0.001

### Feature dimensionality reduction

**Figure 3:**
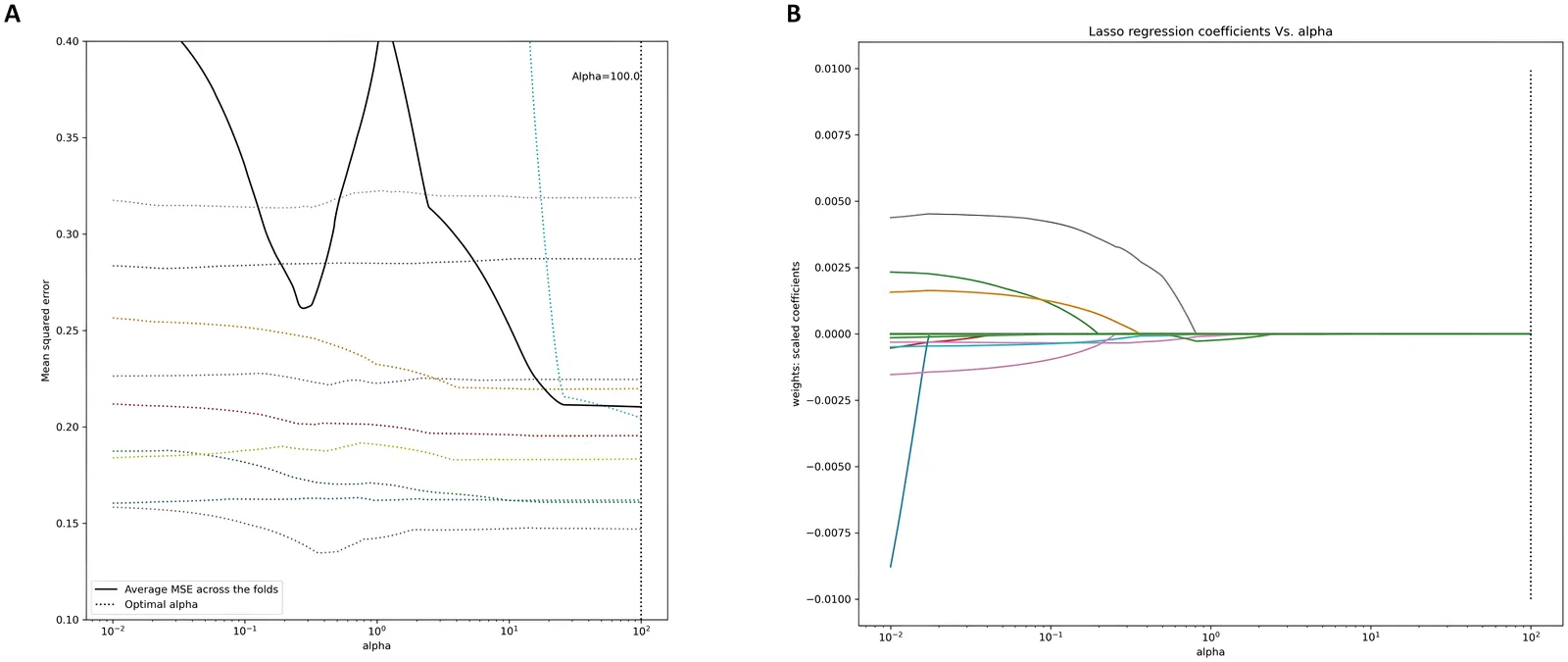
**(A)** The x-axis shows the LASSO penalty parameter α, the y-axis displays the mean-squared error from ten-fold cross-validation, colored dashed curves trace the fold-specific errors, the black solid curve gives their average, and the black dashed vertical line marks the α that minimizes the error. **(B)** With α on the x-axis and coefficient magnitude on the y-axis, colored curves track each feature along the LASSO regularization path, and the black dashed vertical line indicates the coefficient values at the optimal α.

**Figure 4:**
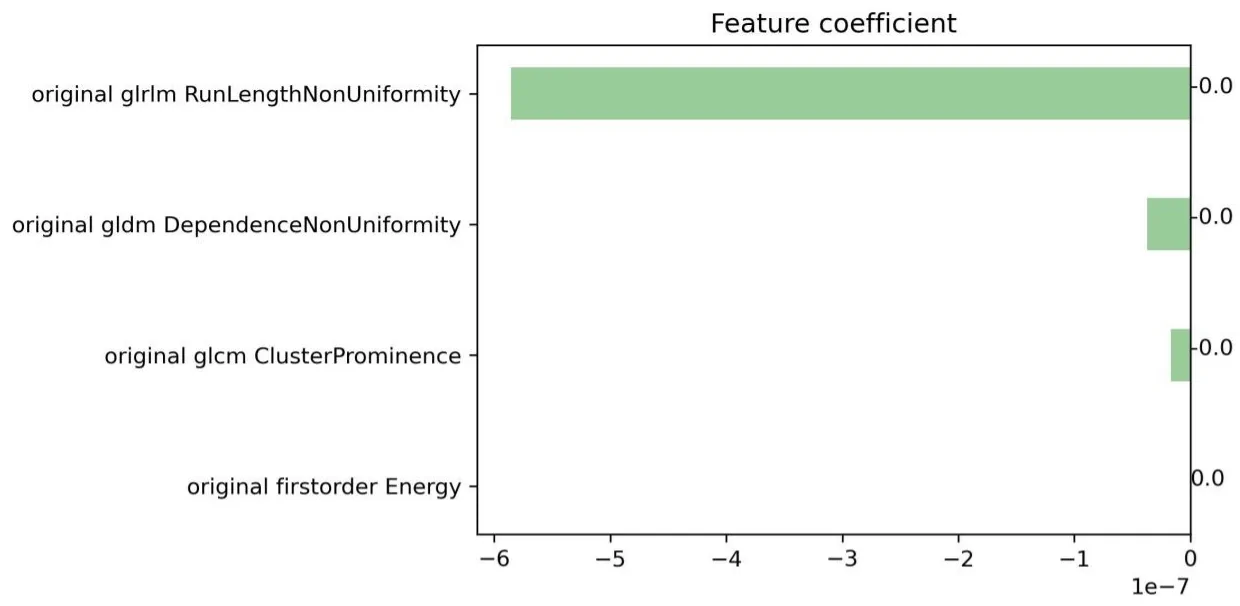
Features retained after the feature selection process for the ADC sequence. Radiomic features from the ADC series that survived selection are ordered on the y-axis, while the x-axis reports their corresponding coefficients.

**Figure 5:**
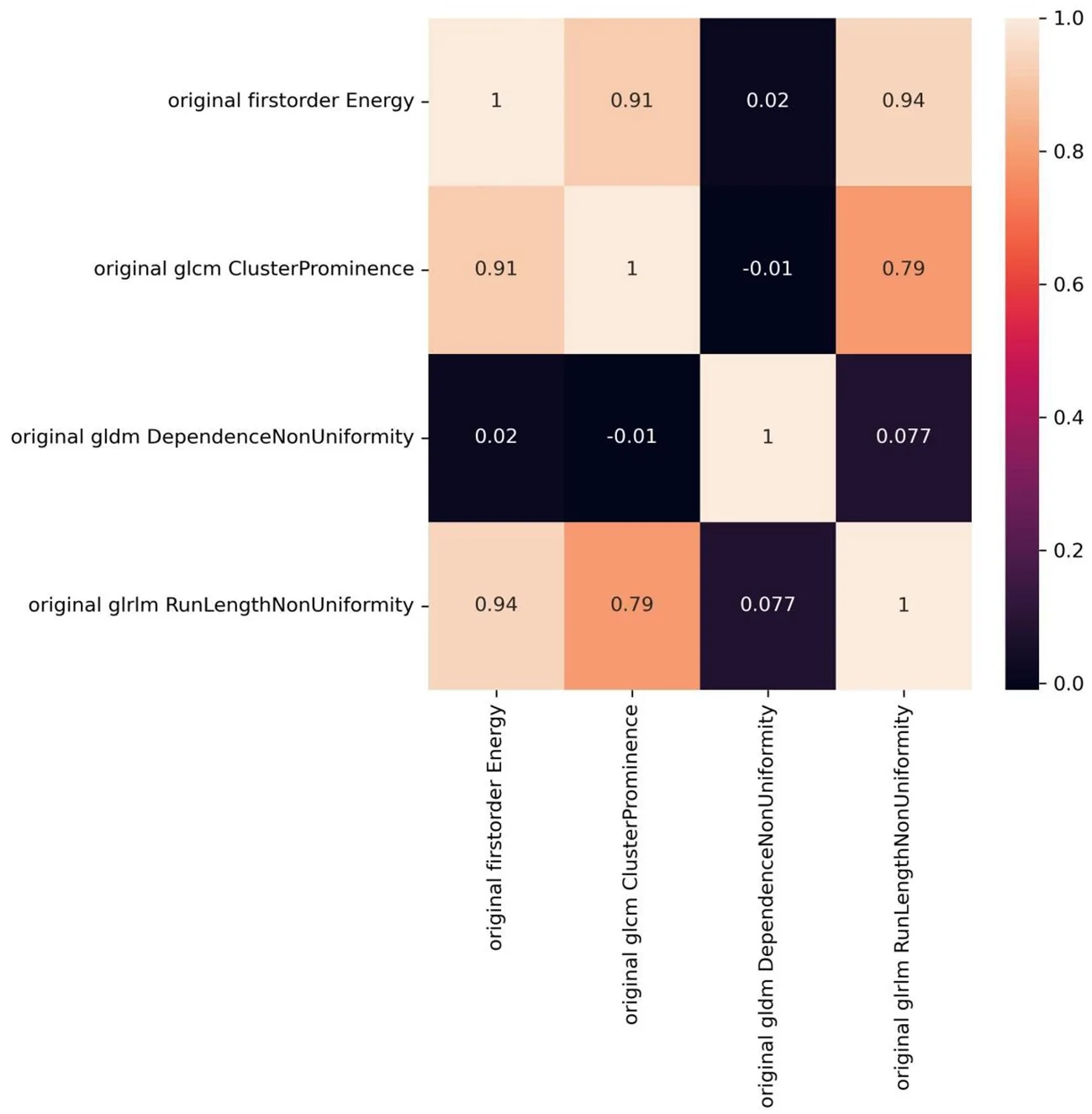
Heat-map of pair-wise correlation among the retained ADC features.

From ADC volumes, 107 radiomic descriptors were extracted following harmonized resampling and z-scoring; wavelet LHL-band variables were discarded *a priori* based on stability considerations (**Figures 3A, B**). A two-stage reduction confined to the training partition yielded a compact four-feature signature consisting of first-order Energy, GLCM Cluster Prominence, GLDM Dependence Non-Uniformity, and GLRLM Run-Length Non-Uniformity (**Figure 4**). Coefficient trajectories along the regularization path and cross-validated error profiles supported the chosen penalty, while pairwise correlation heat maps indicated limited redundancy among the retained variables (**Figure 5**). These features encoded intensity magnitude, higher-order textural asymmetry, and heterogeneity in local dependence and run-length patterns, aligning with pathophysiologic expectations for malignant tissue.

### Model performance

**Figure 6:**
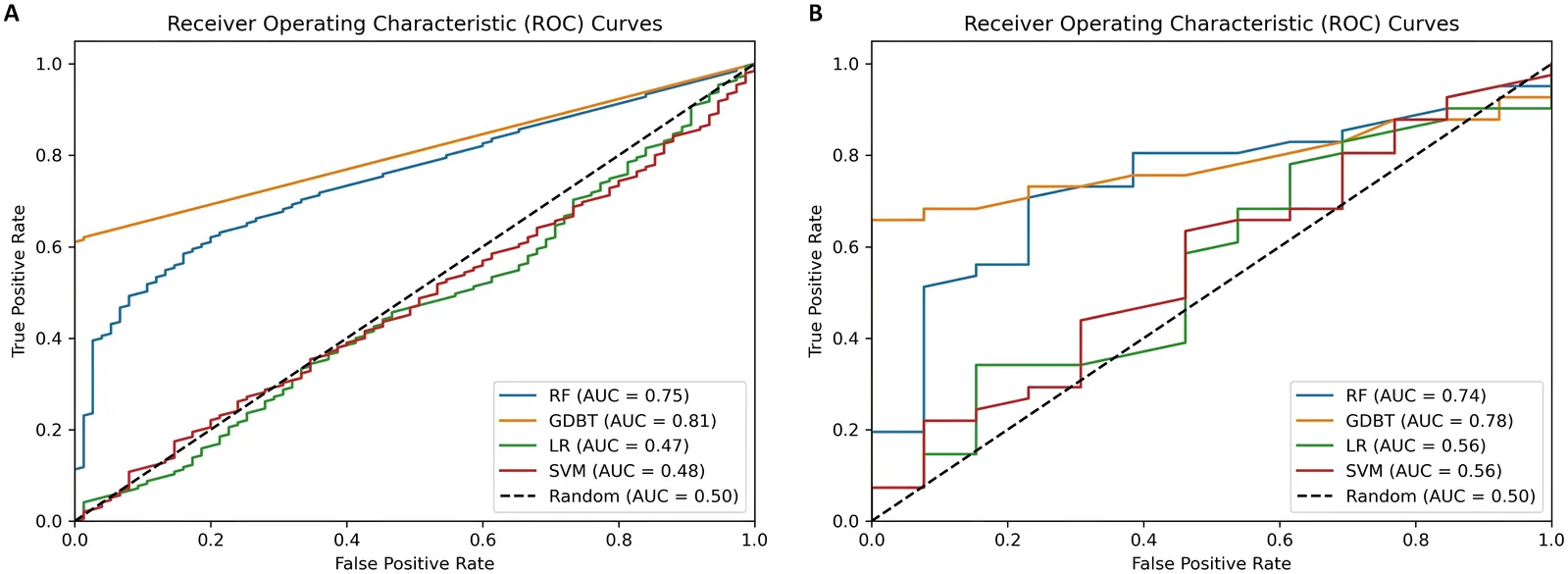
**(A)** The AUC in the training set. **(B)** The AUC in the testing set.

Using this signature, four conventional learners, including LR, SVM with a radial-basis kernel, RF, and GBDT, were trained in the development set and evaluated in the independent test set. Across metrics, GBDT produced the most consistent discrimination. In the training cohort, GBDT achieved an AUC of 0.81 (95% CI: 0.75-0.87, Brier score: 0.11 (95% CI: 0.08-0.15)), with accuracy 0.72 (95% CI: 0.65-0.80), precision 0.73 (95% CI: 0.65-0.81), recall 0.99 (95% CI: 0.98-1.00), F1 score 0.84 (95% CI: 0.75-0.91) and specificity 0.80 (95% CI: 0.71-0.90). Performance remained stable in the test cohort, with an AUC of 0.78 (95% CI: 0.72-0.85, Brier score: 0.15 (95% CI: 0.10-0.21), accuracy 0.76 (95% CI: 0.69-0.84), precision 0.76 (95% CI: 0.65-0.81), recall 1.00 (95% CI: 1.00-1.00), F1 score 0.86 (95% CI: 0.80-0.92) and specificity 0.80 (95% CI: 0.62-0.88). By comparison, the RF model achieved AUCs of 0.75 (95% CI: 0.65-0.84) (training) and 0.74 (95% CI: 0.65-0.87) (testing), while LR and SVM showed more modest AUCs (training: 0.47 (95% CI: 0.29-0.66) and 0.48 (95% CI: 0.22-0.63); testing: 0.56 (95% CI: 0.39-0.72) and 0.56 (95% CI: 0.31-0.78), respectively. **Table 2**, **Figures 6A, B**). The Delong test indicates that all models show statistically significant differences on both the training and validation sets (**Table 3**).

**Table 2:** Performance comparison of all models across training and testing data.

Dataset	Method	Accuracy (95% CI)	Precision (95% CI)	Recall (95% CI)	F1 (95% CI)	Specificity (95% CI)	AUC (95% CI)
Training	GBDT	0.72 (0.65-0.80)	0.73 (0.65-0.81)	0.99 (0.98-1.00)	0.84 (0.75-0.91)	0.80 (0.71-0.90)	0.81 (0.75-0.87)
	LR	0.72 (0.64-0.80)	0.72 (0.63-0.82)	0.99 (0.98-1.00)	0.84 (0.75-0.90)	0.77 (0.69-0.84)	0.47 (0.29-0.66)
	RF	0.72 (0.63-0.80)	0.72 (0.63-0.82)	1.00 (1.00-1.00)	0.84 (0.76-0.90)	0.62 (0.48-0.76)	0.75 (0.65-0.84)
	SVM	0.72 (0.63-0.80)	0.72 (0.63-0.82)	1.00 (1.00-1.00)	0.84 (0.75-0.91)	0.18 (0.05-0.38)	0.48 (0.22-0.63)
Testing	GBDT	0.76 (0.69-0.84)	0.76 (0.65-0.81)	1.00 (1.00-1.00)	0.86 (0.80-0.92)	0.73 (0.62-0.88)	0.78 (0.72-0.85)
	LR	0.74 (0.65-0.81)	0.75 (0.66-0.81)	0.98 (0.96-1.00)	0.85 (0.75-0.91)	0.56 (0.43-0.70)	0.56 (0.39-0.72)
	RF	0.76 (0.66-0.85)	0.76 (0.66-0.82)	1.00 (1.00-1.00)	0.86 (0.75-0.91)	0.52 (0.42-0.62)	0.74 (0.65-0.87)
	SVM	0.76 (0.66-0.85)	0.76 (0.66-0.82)	1.00 (1.00-1.00)	0.86 (0.75-0.91)	0.68 (0.55-0.78)	0.56 (0.31-0.78)

**Table 3:** Delong test comparing LR, RF, and SVM with the GBDT model on the training and validation sets.

Comparison (vs. GBDT)	Training P-value	Testing P-value
LR	<0.001	0.002
RF	0.008	0.003
SVM	<0.001	0.005

ROC curves illustrated these differences at a threshold-free level, with GBDT yielding the largest area under the curve in both partitions. Confusion-matrix patterns reflected the high recall observed for tree-based ensembles, particularly in the test set where false negatives were minimal. Together, these findings indicate that a minimal ADC-only signature can sustain clinically relevant discrimination without reliance on multiparametric inputs or manual lesion delineation, and that gradient boosting offers a favorable bias-variance trade-off for this feature space.

### Auxiliary diagnostic value of the model

**Figure 7:**
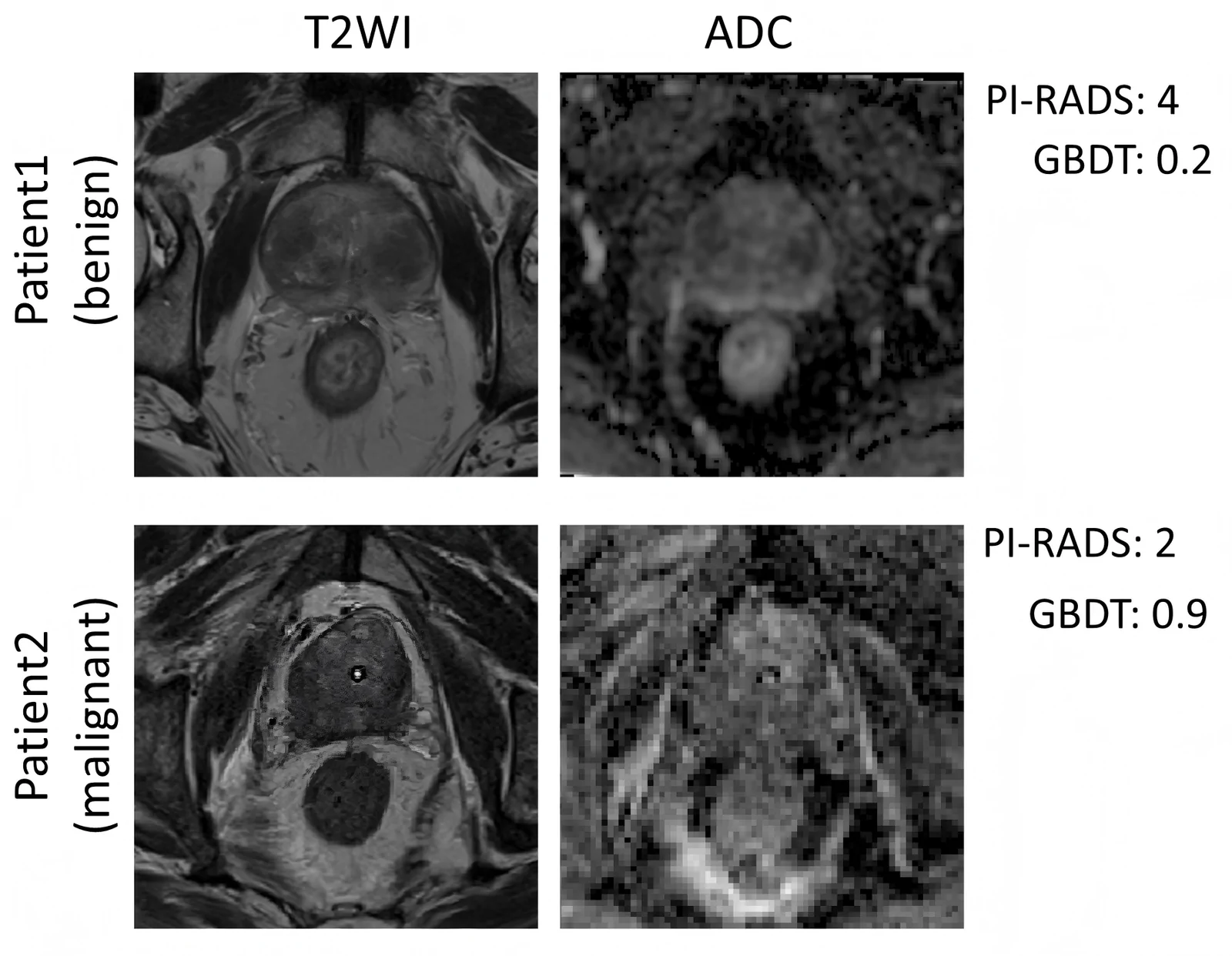
Representative patient cases. PI-RADS indicates the prostate imaging reporting and data system; GBDT denotes the prediction score generated by the best-performing model developed in the present study.

**Figure 7** presents two representative cases. Patient 2 was assigned a PI-RADS score of 2, suggesting a low likelihood of malignancy; however, the GBDT model yielded a high-risk score of 0.9. Subsequent histopathological examination confirmed a malignant diagnosis. In contrast, Patient 1 was classified as PI-RADS 4, while the GBDT model assigned a low-risk score of 0.2, and the lesion was ultimately confirmed to be benign. These findings suggest that GBDT may provide complementary risk stratification beyond conventional clinical assessment, potentially reducing both missed diagnoses and misclassification.

## Discussion

PCa remains one of the most frequently diagnosed cancers in men worldwide (32). Radiomics, as an emerging tool, offers additional value by extracting quantitative features from medical images to enhance diagnostic decision-making (33, 34).

This study demonstrates that a compact, ADC-only radiomics signature paired with a gradient boosting classifier can differentiate malignant from non-malignant prostate lesions with high sensitivity and stable overall discrimination in an independent hold-out cohort. By constraining inputs to a single routinely acquired sequence and reducing the feature set to four stable descriptors, the model delivers clinically relevant performance while minimizing the operational burden that often limits broader radiomics adoption. The observation that gradient boosting consistently outperformed or matched alternative learners aligns with its strength in capturing non-linear interactions among a small set of informative predictors, offering a favorable bias–variance balance for deployment on modest datasets.

The clinical implications are twofold. First, an ADC-only pathway lowers the entry threshold for quantitative support in centers where multiparametric protocols or subspecialty interpretation are not consistently available. Second, the operating profile observed here, near-perfect recall with balanced precision, suits triage use cases in which false negatives are particularly consequential. In practice, such a tool could complement Prostate Imaging Reporting and Data System (PI-RADS) assessment by flagging cases likely to harbor malignancy and by stabilizing decisions in equivocal scenarios, thereby standardizing downstream biopsy and surveillance pathways (35).

The diagnostic utility of ADC-based radiomics has been increasingly recognized in recent literature. Zou et al. developed a hybrid radiomics model combining T2-weighted and ADC imaging features, obtaining AUCs of 0.989, 0.982, and 0.976 in training, testing, and external validation cohorts, respectively, for differentiating clinically significant prostate cancer (csPCa) (36). Similarly, Zhang et al. demonstrated that integrating radiomic derived features from T2-weighted and ADC images improved overall classification accuracy, achieving AUCs of 0.876 in the training set and 0.844 in the validation set (37). Using T2WI and DWI images, Bekou et al. achieved accurate PCa diagnosis through the application of various machine learning methods (16). These results are consistent with our study and confirm the critical role of ADC-based radiomic signatures in enhancing the diagnostic efficacy of machine learning models for PCa.

Methodologically, the results underscore the value of parsimony. Radiomics studies frequently explore extensive, multi-sequence, and sometimes deep-feature spaces; while this can raise apparent accuracy, it also inflates variance and complicates reproducibility. Most of these methods rely on manual tumor segmentation and require the integration of multi-temporal images. The complex segmentation of PCa lesions consumes a significant amount of time for radiologists, and multi-temporal images not only increase the difficulty of data acquisition but also undermine the model’s robustness. Our hierarchical reduction strategy, which combined initial redundancy pruning with sparsity-inducing selection, yielded a four-feature panel spanning first-order intensity (Energy) and texture heterogeneity (Cluster Prominence, Dependence Non-Uniformity, Run-Length Non-Uniformity). The stability and complementary roles of these features are coherent with tumor microarchitecture: malignant glands often exhibit disordered acinar arrangements and stromal remodeling that manifest as asymmetric, non-uniform textural patterns on diffusion-derived maps (38–41). Our pipeline does not require segmentation of PCa or the prostate, and uses only ADC images; it significantly reduces the physician’s workload while maintaining good generalizability.

These results align with those reported by Zhang et al., whose biparametric MRI-based radiomic classifiers distinguished PCa from benign lesions in patients with PSA levels between 4–10 ng/mL and achieved an AUC of 0.844 in the validation cohort, with GLRLM-derived features such as Run Length Non Uniformity contributing substantially to model performance (37). Comparable results were demonstrated by Li et al., whose radiomic analysis of biparametric MRI enabled the differentiation between MR-visible and MR-non-visible clinically significant PCa, with texture features including Dependence Non Uniformity identified as key predictors of lesion visibility (42). Additional support for the predictive value of features such as Cluster Prominence and first-order Energy was provided by Xu et al., whose mpMRI-derived radiomics model for detecting extraprostatic extension in PCa identified these features among the most relevant and achieved high classification performance with robust model generalizability (43).

The consistency of feature selection across these studies indicated that the four features retained in our research, which were derived from ADC sequences alone, can independently reflect tumor-related texture irregularities and diffusion characteristics, and achieve comparable diagnostic performance to models based on multiparametric imaging. These results supported the clinical potential of ADC-only radiomics in PCa diagnosis when combined with appropriate feature selection and classifier design.

Our model differs from previous work in that it achieved high diagnostic accuracy using a limited set of well-curated radiomic features from a single MRI sequence (ADC), rather than relying on multiparametric data. While mpMRI has been shown to improve diagnostic performance (44), our findings suggest that focused feature extraction from ADC sequences alone may suffice in certain contexts, thereby simplifying data acquisition and model implementation. A recent study by Mylona E et al. supports our findings, showing that ADC-derived features had the highest discriminatory power, while T2-weighted imaging features were less informative, and their combination did not improve performance (45).

The consistency in feature selection across these studies suggests that the four features retained in our study (derived solely from the ADC sequence) can independently reflect tumor-related textural irregularities and diffusion properties, and perform comparably to models based on multiparametric imaging in terms of diagnostic accuracy. Our model achieves high diagnostic accuracy using a carefully selected, limited number of radiomic features from a single MRI sequence (ADC), without relying on multiparametric data. Although multiparametric magnetic resonance imaging (mpMRI) has been shown to enhance diagnostic performance (44), our results suggest that, in certain cases, targeted feature extraction from ADC sequences alone is sufficient, thereby simplifying data acquisition and model implementation. A recent study by Mylona E et al. also supports our findings, showing that features derived from ADC exhibit the highest discriminative power, while T2-weighted imaging features provide less information, and combining the two does not improve diagnostic performance (45).

Among the machine learning models based on comprehensive ADC image features, the GBDT model performed best (AUC of 0.81, 95% confidence interval: 0.75–0.87), confirming the effectiveness and applicability of this feature set in prostate cancer diagnosis. Moreover, our model has the potential to provide clinically meaningful decision-support cues for physicians during the diagnostic process. By integrating the GBDT score with established diagnostic frameworks such as PI-RADS, clinicians may obtain a more objective and standardized assessment of disease risk, thereby reducing reliance on subjective interpretation. Such a complementary strategy may enhance diagnostic confidence and facilitate more accurate clinical decision-making. Importantly, the combination of imaging-based risk stratification and model-derived quantitative scores may improve both sensitivity and specificity, ultimately supporting more precise identification of patients and optimizing downstream clinical management.

The study nevertheless has limitations that temper generalization. The single-center, retrospective design constrains demographic and protocol diversity, and although two scanners were included, the range of vendors, coils, and acquisition recipes was far from exhaustive. The reliance on an analysis mask without manual lesion tracing favors scalability but may dilute lesion-specific signals in glands with multifocal disease or diffuse benign changes. Moreover, while an ADC-only strategy simplifies operations, it also forgoes complementary information available from T2-weighted and contrast-enhanced sequences that could enrich discrimination—particularly for borderline cases—if integrated with appropriate guardrails against overfitting. Finally, the current work prioritized discrimination; prospective evaluation of calibration, decision-curve utility, and workflow impact will be essential for clinical adoption.

Future work should pursue multicenter validation with pre-registered protocols that explicitly test robustness to vendor and protocol heterogeneity and report calibration alongside discrimination. Integrating a concise set of clinical covariates—such as age, PSA metrics, or prior biopsy status—may further stabilize performance without sacrificing parsimony. Explainability should be advanced beyond aggregate feature importance toward case-level attributions that help radiologists understand when and why the model diverges from visual impressions, thereby supporting accountable use. Finally, closing the loop with prospective studies that embed the model in the reporting workflow and measure effects on biopsy decisions, time-to-diagnosis, and downstream outcomes will determine whether the observed gains translate into tangible clinical benefit.

In sum, an annotation-light, ADC-only radiomics model built on a compact feature set and gradient boosting achieved high sensitivity and stable overall performance while reducing the complexity that often impedes translation. These characteristics recommend the framework as a pragmatic adjunct to prostate MRI interpretation and a credible starting point for multicenter prospective evaluation and incremental multimodal extensions.

## Data Availability

The original contributions presented in the study are included in the article/supplementary material. Further inquiries can be directed to the corresponding author/s.
